# The Importance of Metamemory Functioning to the Pathogenesis of Psychosis

**DOI:** 10.3389/fpsyg.2017.00304

**Published:** 2017-03-06

**Authors:** Sarah Eisenacher, Mathias Zink

**Affiliations:** ^1^Department of Psychiatry and Psychotherapy, Central Institute of Mental Health, University of Heidelberg/Medical Faculty MannheimMannheim, Germany; ^2^Department of Psychiatry, Psychotherapy and Psychosomatics, District Hospital AnsbachAnsbach, Germany

**Keywords:** memory confidence, metamemory, pathogenetic research, psychosis, schizophrenia

## Abstract

Many studies up to date have implied that biases in the metacognition of memory, so called metamemory, contribute to the development and maintenance of positive symptoms in schizophrenia. However, no study exists which has longitudinally followed patients experiencing positive symptoms. The present article therefore reviews cross-sectional studies on retrospective metamemory abilities in participants within different stages of a schizophrenia spectrum disorder, with heterogeneous symptom severities, creating a pseudo-longitudinal overview. Summarized, a deterioration of these abilities correlating with psychosis development can be inferred. The reviewed publications indicate that metamemory biases can already be found in patients with an at-risk mental state for psychosis (ARMS). Patients in their first episode of psychosis (FEP) seem to be more severely impaired than ARMS-patients but similarly affected compared to chronic patients. The contribution of these biases to the pathogenesis of psychosis is discussed, giving consideration to relations with other cognitive- and metacognitive functions, neurochemical processes and neural correlates. It is hypothesized that the biases represent early cognitive markers of the beginning and persisting psychotic state. An early treatment program could help patients to ameliorate the general course of illness or even to prevent the risk of a transition to psychosis.

## Background

Recent literature has demonstrated a reduced metacognitive capacity in patients with schizophrenia. Metacognition refers to the knowledge and cognition of one's cognitive processes (Flavell, 1979), or “thinking about thinking.” Being aware of one's cognitive functionality and cognitive errors is a necessary condition in order to adequately plan strategies for emerging tasks, evaluate their success and adapt one's approach according to situational requirements. In general, metacognitive abilities are important for several cognitive processes, such as communication, attention, memory, and problem solving (Flavell, 1979).

One example of a metacognitive ability is the metacognition of memory processes, named metamemory. Metamemory includes two aspects: the knowledge about memory processes and their awareness (Flavell, 1979; Moritz et al., 2006c). Metamemory knowledge describes the general knowledge or the beliefs that we have about our memory processes. Metamemory awareness rather corresponds to the monitoring and regulation of memory (Perfect and Schwartz, 2002). As it is generally true for metacognition, metamememory abilities provide a foundation for the recognition of one's memory competences, for the correction of beliefs about these competences if required, and for the adaption of strategies to meet the task requirements. For example, metamemory plays a role in recognizing and correcting false memories in a memory task. Several ways of assessing metamemory functioning have proven to be appropriate. First, self-rating questionnaires, such as the Prospective and Retrospective Memory Questionnaire (PRMQ; Mäntylä et al., 2010), can be used to ask participants about their general insight into their memory ability. Second, asking for introspective judgements about one's current memory performance is a qualified strategy to assess metamemory awareness. These judgements can either be given at the time point of memory acquisition, memory retention or memory retrieval (Nelson and Narens, 1990). Introspective judgements can be assessed either prospectively, thus before the required act is performed (“How efficiently will your acquisition/retrieval be?”), or retrospectively (“How efficient has your acquisition/retrieval been?”). Prospective metamemory judgments at the time of encoding can be assessed using Judgements of Learning (JOL; Bacon et al., 2007), namely evaluations of one's learning quality with respect to an upcoming task. For prospective metamemory judgements at the time of retrieval one may use Feeling of Knowing ratings (FOK; Bacon et al., 2001) which assess one's certainty that an item will be recognized in an upcoming recognition task after it has not been recalled independently. A further standard and valid technique in schizophrenia research is the acquisition of retrospective confidence level ratings (CL) pertaining to memory retrieval. These CL ratings have been studied in several different samples with patients with schizophrenia and over the course of schizophrenia development. Therefore, this review focuses on CL ratings as a measure of metamemory ability. CL ratings have been assessed in a variety of tasks, for example verbal recognition tasks (Kircher et al., 2007), verbal (Moritz et al., 2004; Laws and Bhatt, 2005; Bhatt et al., 2010; Eifler et al., 2015; Eisenacher et al., 2015; Hodgetts et al., 2015) or visual (Moritz et al., 2008; Peters et al., 2012, 2013) false memory tasks, source monitoring tasks (Moritz and Woodward, 2002, 2006a,b; Moritz et al., 2003, 2005; Doré et al., 2007; Garcia et al., 2012) or general knowledge tasks (Bacon et al., 2001).

The importance of metamemory functioning to schizophrenia lies not only in its potential mode of action within the development and maintenance of positive symptoms (Eifler et al., 2015; Eisenacher et al., 2015) but also in its relevance to psychosocial functioning (Scheyer et al., 2014). It is assumed that biases in metamemory are associated with a stronger severity of positive symptoms, particularly with delusions (Moritz and Woodward, 2006a). Furthermore, it is hypothesized that metamemory biases are cognitive markers of the beginning psychotic state (Eisenacher et al., 2015). However, the relation between metamemory abilities and positive symptoms is as yet incompletely understood. Furthermore, whether metamemory biases progress differently across subjects and whether there are subgroups of patients experiencing varying metamemory biases has not been investigated in existent studies. To investigate these topics further, knowledge about metamemory biases across stages of psychosis development is necessary, namely before, during and after the exacerbation of a psychosis. Unfortunately, up to date no longitudinal study focusing this topic exists. Instead, many cross-sectional studies have been conducted. These give insight into metamemory functioning in different phases of psychosis development. The aim of the present review was to establish an overview over metamemory abilities in schizophrenia spectrum disorders as assessed by retrospective confidence level ratings. It was aimed at incorporating the current knowledge received from cross-sectional studies into a pseudo-longitudinal picture of metamemory within the pathogenesis of psychosis, from subclinical participants with high schizotypy scores over at-risk mental state patients to patients with chronic schizophrenia.

## Methods

### Identification of relevant literature

Studies on metamemory performance within the course of psychosis were identified by systematic searches via the electronic data bases PsycInfo, PubMed, MedLine and personal communication. Following search terms were used: “metamemory,” “overconfidence,” “memory confidence,” “knowledge corruption” in combination with “schizo^*^” or “psychosis.” We limited the search to publications written in English or German language.

**Figure 1:**
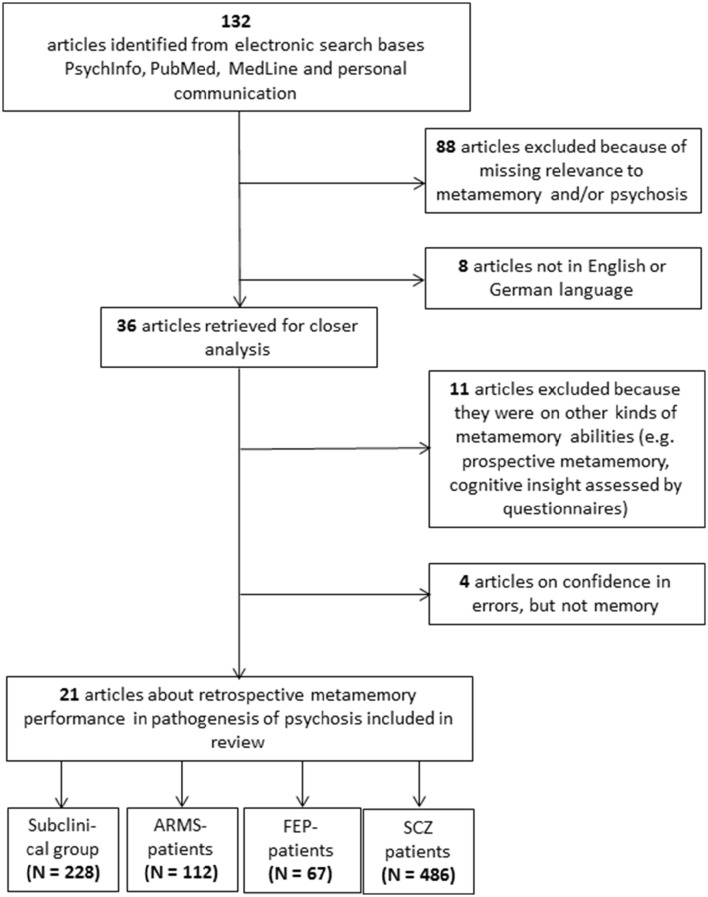
**Consort Flow of the enrollment process**. The last boxes indicate the total summated number of participants over all articles per group. ARMS, At-risk Mental State; FEP, First Episode of Psychosis; SCZ, Schizophrenia.

The literature search yielded a total of 132 papers. A detailed overview of the enrollment process can be found in Figure 1. The included studies were attributed to four different clinical categories and, in a second step, according to the implemented experimental metamemory task. All included studies are summarized in Table 1.

**Table 1:** **Overview of reviewed articles**.

**Studyname**	**Authors**	**Sample**	**Methods**	**Results**
High estradiol levels improve false memory rates and metamemory in highly schizotypal women	Hodgetts et al., 2015	73 women; high (*n* = 37) and low (*n* = 36) estradiol groups	- DRM Paradigm with six word lists - Schizotypy Questionnaire: O-LIFE - Hormone assays by saliva samples	- Less false memories in highly schizotypal participants with high estradiol - Less confidence in false memories in highly compared to those with low estradiol - Low schizotypy participants with high estradiol were more confident by trend
Investigation of metamemory functioning in the at-risk mental state for psychosis	Eisenacher et al., 2015	34 ARMS, 21 FEP, 38 HC	- DRM Paradigm with six word lists - MATRICS MCCB, TMT-B, WCST, MWT-B - Elaborate psychopathological assessment incl. PANSS, SANS, CDSS, PSYRATS, PSP, CGI, GAF, ERIraos, CAARMS	- Lower confidence gap and higher knowledge corruption index FEP-patients compared to ARMS-patients and HC - Lower confidence gap in ARMS-patients compared to HC - Negative association between confidence gap and delusion scores - No association between depressive symptoms and metamemory deficits - Negative association between knowledge corruption index and working memory in the entire patient-group and the ARMS-group
Metamemory in schizophrenia: retrospective confidence ratings interact with neurocognitive deficits	Eifler et al., 2015	32 SCZ, 25 HC	- DRM Paradigm with six word lists - MATRICS MCCB, TMT-B, WCST, MWT-B - Elaborate psychopathological assessment incl. PANSS, SANS, CDSS, PSYRATS, PSP, CGI, GAF	- Lower recognition accuracy, lower confidence gap and higher knowledge corruption index in SCZ - Metamemory biases not correlated to positive symptoms - SCZ impaired in all neuropsychological domains - Knowledge corruption negatively associated with neuropsychological performance in the domains executive function (attention allocation and set-shifting) and working memory - Confidence gap negatively associated with proportion of errors (WCST)
Metacognition in Non-psychotic Help-seeking Adolescents: associations with Prodromal Symptoms, Distress and Psychosocial Deterioration	Scheyer et al., 2014	78 ARMS (age 13–18)	- Two scales of social and role functioning - Non-social (verbal memory, executive functioning) and social (facial emotion perception, ToM) cognition tasks incl. confidence ratings - Prodromal Questionnaire (PQ) - Structured Interview for Prodromal Syndromes (SIPS)	- No difference between ARMS-low risk and ARMS-high risk in levels of cognitive and metacognitive functioning - Metacognitive version improved prediction of psychosocial functioning
Neurocognition and cognitive biases in schizophrenia	Garcia et al., 2012	72 SCZ or schizoaffective disorder	- Source memory task with confidence level ratings - Beck Cognitive Insight Scale - SCID - Verbal learning: HVLT	- Knowledge corruption negatively associated with delayed verbal recall - Cognitive insight not associated with verbal learning and verbal memory
Impact of emotionality on memory and meta-memory in schizophrenia using video sequences	Peters et al., 2012	27 SCZ or schizoaffective disorder, 24 HC	- Emotional video paradigm with subsequent recognition task and confidence ratings - Psychopathology: MINI, PANSS - Premorbid intelligence: MWT-B	- Impaired recognition accuracy in SCZ: more misses, fewer correct items - Less recognitions for negative and neutral video in SCZ, no group difference for positive video - 2x higher knowledge corruption and reduced confidence gap in SCZ - Metamemory performance independent of emotionality - No association between metamemory bias and symptom severity
Investigating the corrective effect of forewarning on memory and meta-memory deficits in schizophrenia patients	Peters et al., 2012	47 SCZ, 47 HC	- Visual variant of DRM paradigm with forewarning instructions - Psychopathology: MINI, PANSS - Premorbid intelligence: MWT-B	- Recognition impairment in SCZ - Reduced confidence gap and increased knowledge corruption in SCZ - No influence of initial forewarning on meta-memory biases
False memory in schizophrenia patients with and without delusions	Bhatt et al., 2010	25 stable chronic SCZ (13 current delusions, 12 without), 20 HC	- DRM Paradigm with eight word lists - Premorbid intelligence: NART - Peter's et al. Delusions Inventory (PDI) - Present State Examination (PSE)	- Delusional group recalled twice as many false-positive memories as HC and non-delusional group - Both patient groups recognized fewer correct words than HC - Both showed greater confidence in their false memories - More false-negative high confidence responses in delusional group than in non-delusional group
Illusions and delusions: relating experimentally-induced false memories to anomalous experiences and ideas	Corlett et al., 2009	50 HC students	- DRM paradigm - Peter's et al. Delusions Inventory (PDI) - Marlowe-Crowne Social Desirability Scale - Chapman Scales	- Higher schizotypy associated with more confidence in errors - Schizotypal symptoms not associated with number of false memories - Schizotypal symptoms correlated with liberal acceptance
Memory and metamemory in schizophrenia: a liberal acceptance account of psychosis	Moritz et al., 2008	68 SCZ or schizoaffective disorder, 25 HC	- Visual metamemory task with figural targets - PANSS - Premorbid intelligence: MWT-B	- False recognition increased in SCZ for weak and moderate lures, similar false recognition for strong lures in both groups - Reduced confidence gap and increased knowledge corruption in SCZ - Negative association between neuroleptic dosage and high-confidence ratings - No correlations between metamemory biases and psychopathology
Metacognition and reflexivity in patients with schizophrenia	Kircher et al., 2007	27 SCZ, 19 HC	- Exp1: verbal Metamemory task - Exp2: description of personality traits - Psychopathology: PANSS, CGI, Schedule for the Assessment of Insight - Neuropsychology: MWT-B, Digit Span Test, Continuous Performance Task	- SCZ show impaired judgment of memory performance - Recognition not different between groups - Patients' ability of personality self-assessment reduced - Memory and metamemory independent - Metamemory no correlated to psychopathology, insight, working memory, attention
Deficient relational binding processes in adolescents with psychosis. Evidence from impaired memory for source and temporal context	Doré et al., 2007	16 FEP-adolescents, 19 HC	- Source monitoring task incl. confidence ratings - PANSS - Wechsler Abbreviated Scale of Intelligence	- FEP more difficulties discriminating target words from neutral - Identification of source and temporal context features worse in FEP - Smaller confidence gap and higher knowledge corruption index in FEP
The contribution of metamemory deficits in schizophrenia	Moritz and Woodward, 2006b	31 SCZ or schizophreniform disorder, 28 PTSD, 20 OCD, 61 HC	- Metamemory: word puzzles/source monitoring -psychopathology: PANSS, MINI	- Decreased confidence gap in SCZ compared to all groups - Higher knowledge corruption in SCZ - Metamemory biases potentially due to liberal acceptance bias - No effect of emotional valence on biases - No correlation between confidence and recognition in SCZ - No correlation between metamemory biases and psychopathology
Investigation of metamemory dysfunctions in fist-episode schizophrenia	Moritz et al., 2006a	30 FEP, 15 HC	- Source memory task (Kent-Rosanoff association test) incl. confidence ratings - PANSS	- Decreased confidence gap and increased knowledge corruption in FEP-patients - More recognition errors in FEP-patients - No correlation between metamemory biases & positive symptoms
Patients with schizophrenia do not produce more false memories than controls but are more confident in them	Moritz et al., 2006c	35 SCZ, 34 HC	- Pictorial recognition task incl. confidence ratings - PANSS, PANADSS	- SCZ impaired on true item recognition - Similar false memories in both groups - Reduced confidence gap in SCZ and increased knowledge corruption index - No correlations between metamemory biases and delusions, suspiciousness, hallucinations
False memories and delusional ideation in normal healthy subjects	Laws and Bhatt, 2005	105 HC undergraduates	- DRM paradigm with eight word lists - Peter's et al. Delusions Inventory	- Poorer recall in high compared to low delusion ideation - More false-alarm memory recalls in high delusion ideation group - Greater confidence for false-positives in high delusion ideation group
Confidence in errors as possible basis for delusions in schizophrenia	Moritz et al., 2005	30 SCZ, 17 HC	- Source memory task (Kent-Rosanoff association test) incl. confidence ratings - Signs and symptoms of psychotic illness scale - Premorbid intelligence: NART	- Greater confidence in errors in SCZ - Knowledge corruption for false-positive and false-negative words - Higher confidence not correlated with delusions - No correlations with IQ
False memories in schizophrenia	Moritz et al., 2004	20 SCZ, 20 HC	- DRM paradigm with six word lists - Signs and symptoms of psychotic illness scale - Premorbid intelligence: NART	- High knowledge corruption for false-negative errors but not false-positive in SCZ - Strong positive correlation between correct recognitions and false-positive recognition of critical lure
Source monitoring and memory confidence in schizophrenia	Moritz et al., 2003	30 SCZ, 21 HC	- Source memory task (Kent-Rosanoff association test) incl. confidence ratings - Brief Psychotic Rating Scale - Premorbid intelligence: MWT-B - Neuropsychology: Rey Auditory Verbal Learning Test, Wisconsin Card Sorting Test	- Deficient source memory and recognition in SCZ - Higher confidence in false answers in SCZ - Correcting effect of neuroleptic dose on confidence gap - No correlations MWT-B, WCST not correlated with metamemory; RAVLT correlated with recognition errors
Memory confidence and false memories in schizophrenia	Moritz and Woodward, 2002	23 SCZ, 15 HC	- Source memory task (Kent-Rosanoff association test) incl. confidence ratings	- High confidence in errors in SCZ - More recognition errors in SCZ - No correlation between knowledge corruption and symptoms - More high confident answers, irrespective of correct and incorrect in patients with current delusions
Consciousness in schizophrenia: a metacognitive approach of semantic memory	Bacon et al., 2001	19 SCZ, 19 HC	- General Knowledge task with retrospective ratings (FOK, CL) - Brief Psychiatric Rating Scale, SAPS, SANS - Wechsler Memory Scale Revised	- No difference in confidence and feeling-of-knowing (FOK) ratings between SCZ and HC - FOK ratings significantly reduced and more frequent discordant in SCZ

*ARMS, at-risk mental state patients; CAARMS, Comprehensive Assessment of At-risk Mental States; CDSS, Calgary Depression Scale for Schizophrenia; CGI, Clinical Global Impressions Scale; DRM, Deese-Roediger-McDermott paradigm; ERIraos, Early Recognition Inventory based on IRAOS; FEP, first episode of psychosis patients; HC, healthy controls; HVLT, Hopkins Verbal Learning Task; IQ, Intelligence Quotient; MATRICS, Measurement and Treatment Research to improve Cognition in Schizophrenia; MCCB, MATRICS Consensus Cognitive Battery; MINI, Mini International Neuropsychiatric Interview; MWT-B, Multiple Choice Word Test, version B; NART, National Adult Reading Test; OCD, Obsessive Compulsive Disorder; PANADSS, Positive and Negative and Disorganized Symptoms Scale; PANSS, Positive and Negative Syndrome Scale; PSP, Personal and Social Performance Scale; PSYRATS, Psychotic Symptom Ratings Scales; PTSD, Posttraumatic Stress Disorder; RAVLT, Rey Auditory Verbal Learning Test; SANS, Scale for the Assessment of Negative Symptoms; SAPS, Scale for the Assessment of Positive Symptoms; SCID, Structured Clinical Interview for DSM; SCZ, schizophrenia patients; ToM, Theory of Mind; TMT-B, Trail Making Test, version B; WCST, Wisconsin Card Sorting Test*.

### Experimental methodologies of the relevant studies

Mainly, two types of tasks have been implemented in the investigation of metamemory abilities using retrospective CL ratings: verbal- or visual false memory tasks and source monitoring tasks. Two further studies used verbal learning tasks (Kircher et al., 2007; Scheyer et al., 2014) and one used a general knowledge task (Bacon et al., 2001).

The standard false memory tasks which were implemented across the different studies were variants of the Deese-Roediger-McDermott (DRM) paradigm (Deese, 1959; Roediger and McDermott, 1995). The task aims at eliciting false-positive errors (i.e., new information is mistaken as having been presented before). Information can either be verbal (word recognition) or visual (picture recognition) in nature. However, the verbal paradigm has been implemented more often (Laws and Bhatt, 2005; Bhatt et al., 2010; Moritz et al., 2014b; Eifler et al., 2015; Eisenacher et al., 2015; Hodgetts et al., 2015). In this version, participants are usually asked to encode lists of words which are strongly associated with a not presented lure or “theme” word. A subsequent recognition task requires the identification of “old/studied” and “new” words. Words that are new but are semantically associated with the theme word are more likely to trigger false-positive answers. By additionally asking for CL ratings, memory monitoring abilities can be assessed (Moritz et al., 2004).

In the standard source monitoring tasks, participants are presented with a wordlist and are required to generate semantic associations for each word. In a recall phase, participants are handed a list of words from different categories: (1) associative words generated by the experimenter; (2) associative words generated by the participant himself; (3) associative or non-associative words which are totally new. The participant is asked to identify whether a word is old or new, determine the source (experimenter or himself) for old words, and indicate the level of confidence for his answer (e.g., Moritz and Woodward, 2002).

Different kinds of indicators have been regarded in the examination of retrospective CL ratings. First of all, the general *confidence in errors* can be measured (Kircher et al., 2007). Going more into detail, Moritz et al. (2003, 2006a) found that patients are usually more confident in erroneous memories while being less confident in correct answers instead. This feature was labeled *decreased confidence gap* and demonstrates an impaired competence in the differentiation between correct and incorrect memories. A third indicator was termed *knowledge corruption index* and expresses the proportion of false answers that are rated as correct with the highest confidence (Moritz et al., 2006a, 2008). Knowledge corruption was found to be excessively high in patients with schizophrenia (Moritz and Woodward, 2006b; Moritz et al., 2008). While memory measures represent the greatest proportion of assessments in this area of research, findings of abberrant confidence ratings are not necessarily bound to memory. Previous studies also confirmed overconfidence for example in visual perception (Moritz et al., 2014a) or mental state perception (Köther et al., 2014).

## Metamemory functioning over the course of illness

### Metamemory in participants with high schizotypy scores

Laws and Bhatt (2005) were the first to investigate metamemory functioning in healthy people using the DRM paradigm. Their results presented the first indications for an association between high delusional scores in healthy people and confidence in memory. High scores on the Peter's et al. delusions inventory (PDI; Peters et al., 2004) were related to higher confidence ratings for false-positive memories. In contrast, participant's confidence in the recognition of studied items was independent of the level of schizotypy (Laws and Bhatt, 2005). Results from a second study with 50 students further confirmed that metamemory biases are not refined to a clinical diagnosis within the schizophrenia spectrum (Corlett et al., 2009). Participants were rated according to their levels of delusional ideation. Confidence in errors in a DRM-task was correlated to self-reported perceptual aberrations as well as to magical thinking on the Peter's et al. delusions inventory. No association was identified between accuracy of memory and delusion proneness measures. A third study by Hodgetts et al. (2015) also aimed at understanding metamemory processes in healthy people with high vs. low levels of schizotypy. The authors found that estradiol had a beneficial effect on metamemory processes which was specific to participants with high schizotypy scores.

**Figure 2:**
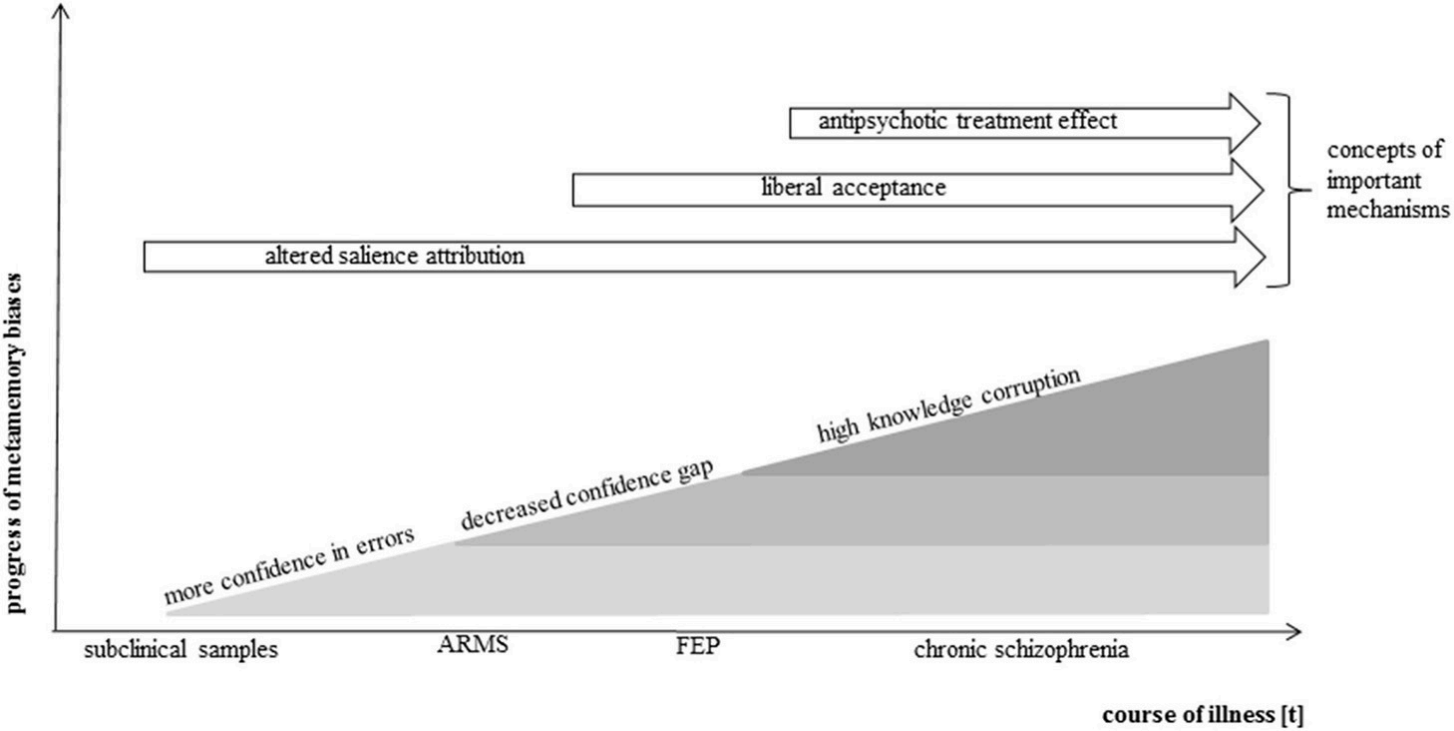
**Simplified model of metamemory in the course of psychosis**. Presented is a modeled progress of metamemory biases along the course of psychosis development. The model is based on the current knowledge gained by above-reviewed metamemory studies in the schizophrenia spectrum. It has been shown that an increased confidence in errors is already present in subclinical samples as a very early metamemory bias. It is assumed that further biases develop with increasing symptom severity and course of illness, complement one another and lead to a growing deterioration of metamemory ability. Antipsychotic treatment may attenuate the effects. Potential cognitive mechanisms are indicated in order of their potential effective time period.

These three studies support the notion that subjects without a clinical diagnosis but with high subclinical scores on delusion-proneness or schizotypy scales are liable to attribute high confidences to memory errors indicating that early delusional symptoms are associated with metamemory processes (Figure 2).

### Metamemory in the at risk mental state for psychosis

In most cases, a prodromal phase, in which pre-psychotic symptoms develop, precedes the acute exacerbation of schizophrenia spectrum disorders (Häfner et al., 2003; Fusar-Poli et al., 2013). As a prospective statement about the development of a psychosis cannot be given definitely when only early symptoms are experienced, this putative prodromal phase is usually called “at risk mental state” for psychosis (ARMS). On average, about 22% of the patients meeting these ARMS-criteria experience a transition to psychosis after 1 year (McGorry et al., 2009; Ruhrmann et al., 2010; Fusar-Poli et al., 2012). The ARMS is mainly characterized by cognitive basic symptoms (BS), attenuated psychotic symptoms (APS) and/or brief limited intermittent psychotic symptoms (BLIPS). Some diagnostic interviews require a generally low or decreasing global functioning, such as the Comprehensive Assessment of At Risk Mental States (CAARMS; Yung et al., 2005). Another comprehensive instrument, the ERIraos (Early Recognition Inventory; Rausch et al., 2013; Maurer et al., 2016), has been used to identify ARMS-samples in studies investigating metacognitive deficits, such as the bias against disconfirmatory evidence (BADE Eisenacher et al., 2016a), the jumping to conclusion bias (JTC; Rausch et al., 2015) and metamemory biases (Eisenacher et al., 2015).

As the pathogenic research in schizophrenia is usually confounded by the specified variables due to the chronic illness course it is reasonable to prevent these confounders by an examination of very early phases of the illness. An even earlier phase than the first psychotic episode is thus the ARMS. Metacognitive deficits in ARMS have been examined rather rarely up to date. Experimental evidence exists for a JTC bias (Rausch et al., 2015, 2016) and some support for a an increasing BADE along the illness process, starting from the ARMS (Eisenacher et al., 2016a), both in comparison to healthy controls. One study exists which examined metamemory functioning in ARMS-patients using the above introduced false memory DRM-paradigm (Eisenacher et al., 2015). In a comparison to healthy control participants, ARMS-patients already exhibited an abberrant metamemory pattern. Similar to first episode patients (see below) they showed a decreased confidence gap. Thus, already at this early stage of symptom development the differentiation between correct and false memories was diminished, demonstrating an early monitoring impairment. However, knowledge corruption of ARMS-patients was not different from healthy controls, in contrast to a comparison between healthy controls and patients with a first episode of psychosis (FEP). A second study by Scheyer et al. (2014) presented no difference in memory monitoring between participants with high and low ARMS-symptoms using an auditory verbal learning task. However, in this study no control group was recruited.

In conclusion, monitoring abilities in the ARMS may be partially spared. Moritz et al. (2008) proposed that monitoring deficits are accompanied by a less thorough search for valid cues leading to less review of essential evidence and reasoning in favor of one's hypothesis. Concurrently, unrecognized errors may occur. This proposition might explain the beginning of psychotic beliefs in the ARMS. It can be hypothesized that a reduced confidence gap in the ARMS reflects a reduced ability to monitor a beginning inordinate belief sufficiently. False beliefs might not be reconsidered but instead accepted with too little search for validation. As a consequence, the risk for errors, wrong reasoning and false conclusions increases. A reduced confidence gap appearing as early as in the ARMS therefore potentially constitutes a basic cognitive deterioration in the formation of delusional beliefs. This deduction is in line with data showing a reduced confidence gap in a visual perception task in healthy controls with high paranoia scores (Moritz et al., 2004).

### Metamemory in early psychosis

The ARMS leads in at least one of five patients to a first manifestation of psychosis (Fusar-Poli et al., 2012). Several studies investigated and detected memory monitoring biases in this early clinical state. Moritz et al. (2006a) explored confidence in memory with a standard source monitoring task in 30 FEP-patients with a diagnosis of schizophrenia or schizophreniform disorder. As hypothesized and in line with prior schizophrenia studies, a decreased confidence gap and an increased knowledge corruption were revealed. The authors concluded that “A reduced distinction between correct and incorrect information in metacognition is proposed to be a vulnerability factor for the development of delusions in schizophrenia” (Moritz et al., 2006a, p. 247). A similar source memory task was employed by Doré et al. (2007) in a group of adolescents with psychotic mood disorders and schizophreniform disorders. Knowledge corruption and the confidence gap were again evaluated. Adolescents showed the identical aberrant monitoring patterns as previously described: they were less able to differentiate between correct and incorrect responses in terms of confidence (decreased confidence gap) and had a higher proportion of high-confident but erroneous answers compared to the healthy control group. A very recent publication of our group (Eisenacher et al., 2015) replicated these findings with a verbal false-memory DRM-paradigm in 21 FEP-patients. The study was able to exclude the typical confounding factors of illness chronicity, e.g., hospitalization or affiliated adaptive behaviors, and long-term antipsychotic medication. Patients were currently untreated regarding antipsychotics and had never been treated for longer than 4 weeks before. Since it has been shown that dopaminergic treatment can influence confidence of judgments (Lou et al., 2011; Andreou et al., 2013) this exclusion of antipsychotic medication is very important for the pathogenic research. All in all, investigations in the early psychosis demonstrated that memory monitoring biases exist in and contribute to this illness phase and are not merely the consequence of long-term illness-related variables (see Figure 2).

### Metamemory in chronic schizophrenia

In a prior review, Moritz and Woodward (2006a) elucidated the topic of memory accuracy and memory monitoring in schizophrenia. They argued that patients with schizophrenia with or without delusions could not be differentiated by memory accuracy, such as a higher number of false-positive errors, but rather by the reference point of confidence in errors. Higher confidences in errors could be found persistently in patients with schizophrenia irrespective of the implemented task design. In contrast to healthy control groups these studies robustly suggested a biased metamemory performance (Moritz and Woodward, 2002; Moritz et al., 2003, 2004, 2005, 2006a; Kircher et al., 2007; Bhatt et al., 2010; Peters et al., 2012, 2013; Eifler et al., 2015), apart from one exception (Bacon et al., 2001). Moritz and Woodward (2002) were the first to compare the previously described source monitoring task between patients with schizophrenia and a healthy control group. Patients attributed higher confidences to memory errors than the control group, thus showing a higher knowledge corruption. Studies from the same research group (Moritz et al., 2003, 2005) replicated these results and expanded their research by exploring confidence in correct answers. They found that confidence in correct answers was indeed lower than in the control group (Moritz and Woodward, 2006b). This reduced differentiation between correct and false answers in terms of confidence was termed reduced confidence gap (Moritz et al., 2003). Similar results were revealed by studies using false memory designs, strengthening prior results. By means of the DRM-paradigm, higher knowledge corruption, especially for false-negative errors (Moritz et al., 2004) as well as a decreased confidence gap (Moritz et al., 2008) were identified in patients with schizophrenia compared to healthy controls. False memory studies that concentrated on different visual stimuli likewise replicated the same metamemory biases (Moritz et al., 2006c, 2008; Peters et al., 2013). It can be supposed that these biases carry some specificity for psychosis because the same differences were found in comparison to clinical samples such as patients with post-traumatic stress disorder or obsessive-compulsive disorder (Moritz and Woodward, 2006b). A later study showed that the biases could especially be found in acute delusional patients in contrast to non-delusional patients (Bhatt et al., 2010). Furthermore, certain cognitive correlates were identified. Poorer delayed verbal recall was associated with an increased knowledge corruption in a source monitoring task (Garcia et al., 2012) and deficits in several neurocognitive measures, executive functioning and working memory in particular, were associated with a decreased confidence gap as well as with an increased knowledge corruption index in a false memory task (Eifler et al., 2015). Examining further influencing factors, it was hypothesized that the initial forewarning of the DRM-effect would decrease metamemory biases. This assumption was, however, not supported (Peters et al., 2012). Likewise, no effect was found of variations in emotional valence (Peters et al., 2013).

Summarized, these studies display consentaneous results on overconfidence in memory errors and a decreased confidence in correct memories in patients with schizophreniform disorders. This knowledge corruption in schizophrenia is a robust finding not only in memory research, but has also been found in visual perception tasks (Moritz et al., 2014a) and mental state perception tasks (Köther et al., 2014; see Figure 2).

## Mechanisms of metamemory biases

### Neurocognition and metamemory

There are ongoing discussions about the mutual influences of metacognitive functioning and its potential basic cognitive associates (Fernandez-Duque et al., 2000). Patients with schizophrenia (Nuechterlein et al., 2004) as well as FEP- and ARMS-patients (Eisenacher et al., 2016b) display multiple neurocognitive deficits, for example concerning the domains executive functioning, working memory, attention and vigilance, problem solving, processing speed, verbal, and visual learning. Impairment in memory accuracy has robustly been found in the above introduced variation of metamemory tasks in chronic patients (Moritz and Woodward, 2002; Moritz et al., 2003, 2006c; Bhatt et al., 2010; Peters et al., 2012, 2013; Eifler et al., 2015). Furthermore, recall-impairment was demonstrated (Garcia et al., 2012). These results were supported by a recent meta-analysis which reported consistent cognitive impairment (Fioravanti et al., 2012). Close interrelations between metacognition and neurocognition have been suggested (Flavell, 1979; Lysaker et al., 2005). From a methodological point of view it can be denied that neurocognitive abilities should be considered basic or primary while metacognitive features appear entirely secondary. Due to a partial overlap of neural structures, networks, and mechanisms (Mäntylä et al., 2010), we would expect significant associations between the assessments of performance in both functional areas. Based on this knowledge, it can be assumed that the assessment of neurocognitive abilities with instruments such as the MATRICS Consensus Cognitive Battery (MCCB; Nuechterlein and Green, 2006) recruits neural mechanisms that are also necessary for performing metamemory tasks.

This assumption is strengthened by correlational analyses. In chronic patients, a higher knowledge corruption index and a lower confidence gap were associated with worse performance in executive functions. A higher knowledge corruption was further associated with less working memory performance (Eifler et al., 2015). An increased knowledge corruption has been associated with poorer delayed verbal recall (Garcia et al., 2012). Similar results have been detected regarding other metacognitive biases, such as the jumping to conclusion bias (Garety et al., 2013; Falcone et al., 2015) and the bias against disconfirmatory evidence (Eifler et al., 2014). It was suggested that executive functioning and working memory are the two main mechanisms for cognitive insight (Orfei et al., 2010). Further indications come from studies regarding neural functioning during cognitive tasks. The prefrontal cortex has been identified as a mediating structure for metacognitive capacities, for the executive system (Fernandez-Duque et al., 2000) as well as for working memory abilties (D'Esposito, 2007). Furthermore, the medial temporal lobe has been found to be involved in cognitive confidences (Moritz et al., 2006b) and working memory tasks (Olsen et al., 2009). A third structure, the cingulate cortex, is involved in metamemory processes (Moritz et al., 2006a; Do Lam et al., 2012; Chen et al., 2013) and in executive functioning, particularly in conflict resolution and conflict monitoring (Fernandez-Duque et al., 2000).

All these findings contrast with a proposition of Kircher et al. (2007) who assumed that metacognition might rather be an autonomous and superordinate system. The different results between studies may be explained by conceptual differences, such as diverse tasks to assess working memory and executive functioning and variations within the metamemory tasks. For example, latencies between learning and recognition within the metamemory task can also affect working memory load. Chua et al. (2009) furthermore stressed the great difficulty to assess meta-level performance without assessing neurocognitive performance in imaging tasks. All in all, metacognitive functioning seems neither to be just a duplicate nor an artifact of neurocognitive functioning as it has been repeatedly emphasized (Veckenstedt et al., 2011; Eifler et al., 2014; Balzan, 2016). Instead, it can be assumed that metamemory and neurocognitive abilities depend on partially overlapping neural networks.

### Neurobiological mechanisms and psychological theories of metamemory

A number of neurobiological and—physiological findings in studies with schizophrenia patients, including structural and functional impairments as well as aberrations in neurotransmission and signaling, give support for the just described procedures. The hippocampus and the prefrontal cortex are two prominent structures in memory processing (Fuster, 2015; Gruart et al., 2015). Structural investigations showed that volume reductions in schizophrenia are evident in the hippocampus (Heckers and Konradi, 2010) and the prefrontal cortex (Ohtani et al., 2014). Using electrophysiological measures, synchronized firing of both structures was found in mouse models of schizophrenia, giving some evidence for an interaction. This interplay was related to cognitive impairment (Dickerson et al., 2010). Firing patterns were found mono-directional and bi-directional, indicating functional connectivity in bottom-up and top-down processes. Imaging studies in humans and in animal models furthermore demonstrated that deficits in memory processes are particularly associated with functional impairment in the interaction between these two structures (for a review see Sigurdsson and Duvarci, 2016). A reduced interaction was demonstrated for task-related activity (being associated with worse performance on the task; Henseler et al., 2010) as well as for resting state activity (Zhou et al., 2008). Interestingly, the reduction is not as concise in ARMS—compared to FEP-patients, however, already in those patients a reduced connectivity between the medial temporal lobe and structures associated with auditory-language and visual-imagery processing was found, hinting toward a developmental functional deterioration (Haut et al., 2015). In summary, the hippocampus and the frontal cortex are involved in connected neural activity which may mirror the procedures necessary for basic memory and higher level meta-memory functioning. Next to these interaction patterns, there is evidence for important activity clusters which are distinct and specific for the meta-level of performance, such as greater activities in medial prefrontal, medial parietal, and lateral parietal regions (Chua et al., 2009).

Using imaging methods, it could repeatedly be found that the just described abnormal hippocampal-frontal connectivity seems to be related to dopaminergic mechanisms (Slifstein et al., 2015; Sigurdsson and Duvarci, 2016). Dopamine furthermore interacts closely with glutamate in the brain and both are in turn related to working memory deficits and other cognitive dysfunctions (Meisenzahl et al., 2007; Salavati et al., 2015). A “large amount” of evidence indicates that metamemory biases are also associated with deficient dopaminergic processes. For example, Lou et al. (2011) administered pergolide, a dopaminergic agonist, to healthy participants in order to construct a model of psychosis-like awareness. They found that pergolide led to increases in confidence. In turn, anti-dopaminergic treatment with the first generation antipsychotic haloperidol decreased the number of highly-confident incorrect answers in healthy controls (Andreou et al., 2013). The concept of aberrant attribution of salience is closely linked with dopaminergic processes (Andreou et al., 2013). It describes that the relevance of a neutral stimulus changes whenever the salience, attributed to this stimulus, is altered. Changes can occur on several interacting dimensions and differentially over time. Within a simplified model, the relevance of increased salience for schizophrenia has been presented (Kapur, 2003). Higher salience attribution and an increase in the importance of a stimulus consequently lead to decisions which are made with heightened confidence (Kapur, 2003; Speechley et al., 2010; Esslinger et al., 2013). The high confidence in a decision therefore dissociates from the objective correctness of this decision. Along the same line, Heinz and Schlagenhauf (2010) hypothesized that dopaminergic processes modulate the attention to conditioned cues and thereby predict reward. A high-confident acceptance might thus operate as a rewarding experience. As a consequence, judgements might be steadily over-interpreted as correct, forming the basis for delusion development (Andreou et al., 2013). If altered salience attribution is not yet strongly pronounced, as it might be the case, for example, in ARMS-patients, an attribution of high salience may not directly lead to high-confident decisions. It may, however, already lead to less differentiation between correct and incorrect memories. Considering this, the aberrant salience attribution theory is also in line with findings of metamemory biases in ARMS-patients and psychosis prone healthy individuals.

Two mechanisms of aberrant salience have been hypothesized, but both may also interact or describe processes from slightly different point of views: it could either result from a deficit in correctly updating contextual information or a disturbed feedback processing within the reward system (Andreou et al., 2013). An updating deficit might be due to an impairment in the inhibition of active information diminishing the capacity for newly incoming information (Orenes et al., 2012). Again, altered activity within the dopaminergic system is a probable contributor, being associated with deficits in gating and the inhibition of information (Cohen and Servan-Schreiber, 1992; Cohen et al., 1996; Perlstein et al., 2001). The mechanism of an erroneous feedback processing is explained by the theory of disturbed error prediction signaling (Fletcher and Frith, 2009). The theory holds that aberrant salience develops through a failure to update one's beliefs in the presence of ambiguous information. It is suggested that strong mismatches between ambiguous information and one's existing expectations cannot be solved at lower cognitive levels and must be pushed upwards the cognitive hierarchy, thereby reaching inappropriate levels of importance. This salience attribution to fragments of information can make patients with schizophrenia vulnerable to a liberal acceptance, namely to a tendency to take this fragmentary information as a sufficient basis for high-confident judgments without taking additional information into account (Moritz and Woodward, 2004). This procedure may increase their vulnerability to accept even implausible alternatives with high confidences leading to high-confident false judgments.

## How do metamemory biases contribute to the pathogenesis of psychosis?

Cognitive models of delusions assume that metamemory biases play a role in the pathogenesis of psychosis, contributing to the emergence and maintenance of positive symptoms (Garety and Freeman, 1999; Garety et al., 2005; Moritz and Woodward, 2006a). The reviewed results revealed that altered metamemory performance indeed accompanies the progression of psychosis. Higher confidence in errors, lower confidence in correct answers and a high knowledge corruption have been found in healthy people with delusional ideation, in ARMS-patients, in patients with a first episode of psychosis and in chronic patients with schizophrenia. Group comparisons demonstrated that metamemory biases aggravate over the course of illness, being less distinct in risk constellations of psychosis but growing stronger with the exacerbation of the first episode (Eisenacher et al., 2015).

Studies which analyzed correlations between overconfidence and delusion severity reported medium to large effect sizes. For example, subclinical participants with high delusional scores or schizotypal traits showed greater confidence in memory errors than those with low scores (Laws and Bhatt, 2005; Corlett et al., 2009). In contrast to healthy people ARMS-patients showed a higher knowledge corruption which was still less distinct than in FEP-patients, who showed the most aberrations (Eisenacher et al., 2015). In the same study, the confidence gap was correlated with the PANSS positive symptom subscore, a single delusional item as well as the PSYRATS items for “delusional conviction” and “life disruption by delusions” with medium effect sizes. Patients with chronic schizophrenia and current delusions were shown to be more confident, regardless of the correctness of their answers (Moritz and Woodward, 2002). In another study, an assocation between delusion severity and confidence was limited to false-negative judgements (Bhatt et al., 2010). Contradicting these results, other investigations (e.g., Moritz et al., 2003, 2004, 2005; Moritz and Woodward, 2006b; Kircher et al., 2007; Peters et al., 2013; Eifler et al., 2015) did not reveal significant associations between positive symptoms and metamemory biases. These studies rather suggested that metamemory biases are independent of delusional symptoms and already present before the onset of severe psychosis, potentially representing early markers of the psychotic state.

In sum, it is reasonable to assume that metamemory biases are at least partially independent of the severity of psychosis symptoms and outlast acute positive symptoms. Furthermore, they seem to be independent from the onset of frank psychosis because the reviewed results confirm early memory monitoring impairment in risk states of psychosis. However, different metamemory indexes seem to be differentially pronounced between the stages of psychosis (Eisenacher et al., 2015). People in risk constellations may already insufficiently monitor a beginning inordinate belief, making them prone to follow unrecognized errors, to insufficiently reconsider these errors and to accept beliefs with too little search for further validation in favor of their own hypothesis (Moritz et al., 2008). This reasoning bias may therefore be the foundation of the development of inordinate beliefs. An increased knowledge corruption, however, may not emerge until patients suffer from an acute psychotic symptomatology (Eisenacher et al., 2015). Moritz and Woodward (2004) postulated that the ability to differentiate between correct and false contributes to knowledge corruption. Possibly, ARMS-patients do not (yet) liberally attribute highest confidences to memory errors because they benefit from a higher clinical insight than FEP-patients and rather try to compensate for deficits. Instead, FEP- and chronic-patients display a distinct knowledge corruption and evaluate their potentially false memories with a strong conviction to be correct (Moritz et al., 2006a; Garcia et al., 2012; Eisenacher et al., 2015). Acute stages might furthermore be reflected by a tendency to generally attribute overconfidence to memories, independent of their correctness (Moritz and Woodward, 2002). Supporting this hypothesis, Eisenacher et al. (2015) found that the confidence gap in acutely psychotic FEP-patients was mainly due to a generally higher confidence in answers, being indeed more concise for errors but also visible for correct answers. All in all, knowledge corruption might contribute to a consolidation of delusional beliefs.

## Limitations

There has been a substantial increase in the number of publications on metamemory performance in the psychosis pathogenesis, enhancing our knowledge about the biases' mediating role over time. But what do we not know yet? Up to date, there has not been any longitudinal study exploring the development of metamemory biases over time. Especially, there is no data available which links the pre-psychotic state with the exacerbation of a first psychosis. Monitoring ARMS-patients until a subgroup transits to psychosis would be the ideal way to understand the development of metamemory performance. Longitudinal data would allow deciding whether metamemory aberrations can predict delusion development. Furthermore, they would provide a better understanding of whether metamemory biases progress in different ways across subgroups of patients or whether different subgroups experience variant forms of the bias throughout.

It is important to be attentive to differences in study populations. Several studies included patients with schizoaffective disorders in addition to schizophrenia. In most studies patients with schizophrenia were medicated with antipsychotic medication. Some studies also allowed augmentation with anxiolytic treatment or other forms of polypharmacy. In some articles medication was not even indicated (e.g., Kircher et al., 2007). As it has been outlined, dopaminergic processes seem to play an important part in explanations of metamemory aberrations (Andreou et al., 2013) rendering any form of antipsychotic treatment a major confound. Studies must certainly control for this covariate. In research of our group, only patients with a monotherapy of antipsychotic agents were included to reach this goal (Eifler et al., 2015). ARMS- and FEP-patients were totally untreated regarding antipsychotic medication (Eisenacher et al., 2015). Medication might have also contributed to lower scores of symptom severity in some studies and thereby could have masked potential associations with metacognitive performance. Moreover, the chronic course of illness may have confounded results in patients with multiple episodes of schizophrenia. Indeed, a matched comparison of ARMS-patients or FEP-patients with patients with chronic schizophrenia is complex to set up due to frequent age- and educational differences as a natural consequence of illness development and has not been published up to date.

## Conclusions and perspectives

Taken together, the here reviewed results foster the theory that metamemory biases constitute basic cognitive deteriorations and potential cognitive markers for the emerging psychotic state. This knowledge is not only important for further research about metacognitive biases within the pathogenesis of psychosis but also provides relevant clinical implications. Prior research was able to demonstrate that treatment of metamemory biases, such as the metacognitive training for schizophrenia (Moritz et al., 2011) is effective in reducing metacognitive biases. In an individualized manner, this approach is currently about to be improved (Schneider et al., 2016). Furthermore, a decreasing effect on delusional severity in patients who only partially responded to antipsychotic medication has been demonstrated (Favrod et al., 2014). Regarding the reviewed results, it seems reasonable to apply (meta-) cognitive treatments already to ARMS patients. Early treatment options can reduce subjective strain and symptom severity and possibly even prevent a transition to a fist psychotic episode.

## Author contributions

Both authors contributed substantially to the manuscript in conception, acquisition and interpretation of the data, in drafting and revising the work and in the final approval of the manuscript. Both authors are accountable for all aspects of the work.

## Funding

MZ was funded by the Deutsche Forschungsgesellschaft (DFG, http://www.dfg.de, projects ZI1253/3-1, ZI1253/3-2). SE was supported by a Grant of Heidelberg University (Landesgraduiertenförderungsgesetz). The funders had no role in study preparation and realization or in the decision to publish.

### Conflict of interest statement

MZ received unrestricted scientific grants of the German Research Foundation (DFG), and Servier; further speaker and travel grants were provided from Pfizer Pharma GmbH, Bristol Myers Squibb Pharmaceuticals, Otsuka, Servier, Lundbeck, Janssen Cilag, Roche, Ferrer, and Trommsdorff. The other author declares that the research was conducted in the absence of any commercial or financial relationships that could be construed as a potential conflict of interest.

## References

[B1] Andreou C. Moritz S. Veith K. Veckenstedt R. Naber D. (2013). Dopaminergic modulation of probabilistic reasoning and overconfidence in errors: a double-blind study. Schizophr. Bull. 40, 558–565. 10.1093/schbul/sbt064 23661634 PMC3984513

[B2] Bacon E. Danion J. M. Kauffmann-Muller F. O. Bruant A. S. (2001). Consciousness in schizophrenia: a metacognitive approach to semantic memory. Conscious. Cogn. 10, 473–484. 10.1006/ccog.2001.0519 11790037

[B3] Bacon E. Izaute M. Danion J. M. (2007). Preserved memory monitoring but impaired memory control during episodic encoding in patients with schizophrenia. J. Int. Neuropsychol. Soc. 13, 219–227. 10.1017/S1355617707070245 17286879

[B4] Balzan R. P. (2016). Overconfidence in psychosis: the foundation of delusional conviction? Cogent Psychol. 3:1135855 10.1080/23311908.2015.1135855

[B5] Bhatt R. Laws K. R. McKenna P. J. (2010). False memory in schizophrenia patients with and without delusions. Psychiatry Res. 178, 260–265. 10.1016/j.psychres.2009.02.006 20466436

[B6] Chen J. Feng T. Shi J. Liu L. Li H. (2013). Neural representation of decision confidence. Behav. Brain Res. 245, 50–57. 10.1016/j.bbr.2013.02.004 23415909

[B7] Chua E. F. Schacter D. L. Sperling R. A. (2009). Neural correlates of metamemory: a comparison of feeling-of-knowing and retrospective confidence judgments. J. Cogn. Neurosci. 21, 1751–1765. 10.1162/jocn.2009.21123 18823230 PMC2709699

[B8] Cohen J. D. Servan-Schreiber D. (1992). Context, cortex, and dopamine: a connectionist approach to behavior and biology in schizophrenia. Psychol. Rev. 99, 45–77. 10.1037/0033-295X.99.1.45 1546118

[B9] Cohen J. D. Braver T. S. O'Reilly R. C. (1996). A computational approach to prefrontal cortex, cognitive control and schizophrenia: recent developments and current challenges. Philos. Trans. R. Soc. Lond. B Biol. Sci. 351, 1515–1527. 10.1098/rstb.1996.0138 8941963

[B10] Corlett P. R. Simons J. S. Pigott J. S. Gardner J. M. Murray G. K. Krystal J. H.. (2009). Illusions and delusions: relating experimentally-induced false memories to anomalous experiences and ideas. Front. Behav. Neurosci. 3, 1–9. 10.3389/neuro.08.053.2009 19956402 PMC2786301

[B11] Deese J. (1959). On the prediction of occurence of particular verbal intrusions in immediate recall. J. Exp. Psychol. 58, 17–22. 10.1037/h0046671 13664879

[B12] D'Esposito M. (2007). From cognitive to neural models of working memory. Philos. Trans. R. Soc. Lond. B Biol. Sci. 362, 761–772. 10.1098/rstb.2007.2086 17400538 PMC2429995

[B13] Dickerson D. D. Wolff A. R. Bilkey D. K. (2010). Abnormal long-range neural synchrony in a maternal immune activation animal model of schizophrenia. J. Neurosci. 30, 12424–12431. 10.1523/JNEUROSCI.3046-10.2010 20844137 PMC6633437

[B14] Do Lam A. T. Axmacher N. Fell J. Staresina B. P. Gauggel S. Wagner T.. (2012). Monitoring the mind: the neurocognitive correlates of metamemory. PLoS ONE 7:e30009. 10.1371/journal.pone.0030009 22242196 PMC3252366

[B15] Doré M. C. Caza N. Gingras N. Rouleau N. (2007). Deficient relational binding processes in adolescents with psychosis: evidence from impaired memory for source and temporal context. Cogn. Neuropsychiatry 12, 511–536. 10.1080/13546800701614098 17978937

[B16] Eifler S. Rausch F. Schirmbeck F. Veckenstedt R. Englisch S. Meyer-Lindenberg A.. (2014). Neurocognitive capabilities modulate the integration of evidence in schizophrenia. Psychiatry Res. 219, 72–78. 10.1016/j.psychres.2014.04.056 24880580

[B17] Eifler S. Rausch F. Schirmbeck F. Veckenstedt R. Mier D. Esslinger C.. (2015). Metamemory in schizophrenia: retrospective confidence ratings interact with neurocognitive deficits. Psychiatry Res. 225, 596–603. 10.1016/j.psychres.2014.11.040 25530415

[B18] Eisenacher S. Rausch F. Ainser F. Englisch S. Becker A. Mier D.. (2016b). Early cognitive basic symptoms are accompanied by neurocognitive impairment in patients with an ‘at-risk mental state’ for psychosis. Early Interv. Psychiatry. [Epub ahead of print]. 10.1111/eip.12350 27169782

[B19] Eisenacher S. Rausch F. Ainser F. Mier D. Veckenstedt R. Schirmbeck F.. (2015). Investigation of metamemory functioning in the at-risk mental state for psychosis. Psychol. Med. 45, 3329–3340. 10.1017/S0033291715001373 26201365

[B20] Eisenacher S. Rausch F. Mier D. Fenske S. Veckenstedt R. Englisch S.. (2016a). Bias against disconfirmatory evidence in the at-risk mental state and during psychosis. Psychiatry Res. 238, 242–250. 10.1016/j.psychres.2016.02.028 27086240

[B21] Esslinger C. Braun U. Schirmbeck F. Santos A. Meyer-Lindenberg A. Zink M.. (2013). Activation of midbrain and ventral striatal regions implicates salience processing during a modified beads task. PLoS ONE 8:e58536. 10.1371/journal.pone.0058536 23484034 PMC3590224

[B22] Falcone M. A. Murray R. M. Wiffen B. D. O'Connor J. A. Russo M. Kolliakou A.. (2015). Jumping to conclusions, neuropsychological functioning, and delusional beliefs in first episode psychosis. Schizophr. Bull. 41, 411–418. 10.1093/schbul/sbu104 25053654 PMC4332946

[B23] Favrod J. Rexhaj S. Bardy S. Ferrari P. Hayoz C. Moritz S.. (2014). Sustained antipsychotic effect of metacognitive training in psychosis: a randomized-controlled study. Eur. Psychiatry 29, 275–281. 10.1016/j.eurpsy.2013.08.003 24176646

[B24] Fernandez-Duque D. Baird J. A. Posner M. I. (2000). Executive attention and metacognitive regulation. Conscious. Cogn. 9, 288–307. 10.1006/ccog.2000.0447 10924249

[B25] Fioravanti M. Bianchi V. Cinti M. E. (2012). Cognitive deficits in schizophrenia: an updated metanalysis of the scientific evidence. BMC Psychiatry 12:64. 10.1186/1471-244X-12-64 22715980 PMC3528440

[B26] Flavell J. H. (1979). Metacognition and cognitive monitoring: a new area of cognitive developmental inquiry. Am. Psychol. 34, 906–911. 10.1037/0003-066X.34.10.906

[B27] Fletcher P. C. Frith C. D. (2009). Perceiving is believing: a Bayesian approach to explaining the positive symptoms of schizophrenia. Nat. Rev. Neurosci. 10, 48–58. 10.1038/nrn2536 19050712

[B28] Fusar-Poli P. Bonoldi I. Yung A. R. Borgwardt S. Kempton M. J. Valmaggia L.. (2012). Predicting psychosis: Meta-analysis of transition outcomes in individuals at high clinical risk. Arch. Gen. Psychiatry 69, 220–229. 10.1001/archgenpsychiatry.2011.1472 22393215

[B29] Fusar-Poli P. Borgwardt S. Bechdolf A. Addington J. Riecher-Rossler A. Schultze-Lutter F.. (2013). The psychosis high-risk state: a comprehensive state-of-the-art review. JAMA Psychiatry 70, 107–120. 10.1001/jamapsychiatry.2013.269 23165428 PMC4356506

[B30] Fuster J. (2015). The Prefrontal Cortex. New York, NY: Academic Press.

[B31] Garcia C. P. Sacks S. A. Weisman de Mamani A. G. (2012). Neurocognition and cognitive biases in schizophrenia. J. Nerv. Ment. Dis. 200, 724–727. 10.1097/NMD.0b013e3182614264 22850310

[B32] Garety P. A. Freeman D. (1999). Cognitive approaches to delusions: a critical review of theories and evidence. Br. J. Clin. Psychol. 38, 113–154. 10.1348/014466599162700 10389596

[B33] Garety P. A. Freeman D. Jolley S. Dunn G. Bebbington P. E. Fowler D. G.. (2005). Reasoning, emotions, and delusional conviction in Psychosis. J. Abnorm. Psychol. 114, 373–384. 10.1037/0021-843X.114.3.373 16117574

[B34] Garety P. Joyce E. Jolley S. Emsley R. Waller H. Kuipers E.. (2013). Neuropsychological functioning and jumping to conclusions in delusions. Schizophr. Res. 150, 570–574. 10.1016/j.schres.2013.08.035 24075604 PMC3824078

[B35] Gruart A. Leal-Campanario R. López-Ramos J. C. Delgado-García J. M. (2015). Functional basis of associative learning and its relationships with long-term potentiation evoked in the involved neural circuits: lessons from studies in behaving mammals. Neurobiol. Learn. Mem. 124, 3–18. 10.1016/j.nlm.2015.04.006 25916668

[B36] Häfner H. Maurer K. Löffler W. van der Heiden W. Hambrecht M. Schultze-Lutter F. (2003). Modeling the early course of schizophrenia. Schizophr. Bull. 29, 325–340. 10.1093/oxfordjournals.schbul.a007008 14552507

[B37] Haut K. M. van Erp T. G. Knowlton B. Bearden C. E. Subotnik K. Ventura J.. (2015). Contributions of feature binding during encoding and functional connectivity of the medial temporal lobe structures to episodic memory deficits Across the prodromal and first-episode phases of schizophrenia. Clin. Psychol. Sci. 3, 159–174. 10.1177/2167702614533949 25750836 PMC4349206

[B38] Heckers S. Konradi C. (2010). Hippocampal pathology in schizophrenia. Curr. Top. Behav. Neurosci. 4, 529–553. 10.1007/7854_2010_43 21312412

[B39] Heinz A. Schlagenhauf F. (2010). Dopaminergic dysfunction in schizophrenia: salience attribution revisited. Schizophr. Bull. 36, 472–485. 10.1093/schbul/sbq031 20453041 PMC2879696

[B40] Henseler I. Falkai P. Gruber O. (2010). Disturbed functional connectivity within brain networks subserving domain-specific subcomponents of working memory in schizophrenia: relation to performance and clinical symptoms. J. Psychiatr. Res. 44, 364–372. 10.1016/j.jpsychires.2009.09.003 19837416

[B41] Hodgetts S. Hausmann M. Weis S. (2015). High estradiol levels improve false memory rates and meta-memory in highly schizotypal women. Psychiatry Res. 229, 708–714. 10.1016/j.psychres.2015.08.016 26292620

[B42] Kapur S. (2003). Psychosis as a state of aberrant salience: a framework linking biology, phenomenology, and pharmacology in schizophrenia. Am. J. Psychiatry 160, 13–23. 10.1176/appi.ajp.160.1.13 12505794

[B43] Kircher T. T. J. Koch K. Stottmeister F. Durst V. (2007). Metacognition and reflexivity in patients with schizophrenia. Psychopathology 40, 254–260. 10.1159/000101730 17440288

[B44] Köther U. Veckenstedt R. Vitzthum F. Roesch-Ely D. Pfueller U. Scheu F.. (2014). Don't give me that look – overconfidence in false mental state perception in schizophrenia. Psychiatry Res. 196, 1–8. 10.1016/j.psychres.2012.03.004 22482796

[B45] Laws K. R. Bhatt R. (2005). False memories and delusional ideation in normal healthy subjects. Pers. Individ. Dif. 39, 775–781. 10.1016/j.paid.2005.03.005

[B46] Lou H. C. Skewes J. C. Thomsen K. R. Overgaard M. Lau H. C. Mouridsen K.. (2011). Dopaminergic stimulation enhances confidence and accuracy in seeing rapidly presented words. J. Vis. 11:15. 10.1167/11.2.15 21346001

[B47] Lysaker P. H. Davis L. W. Lightfoot J. Hunter N. Stasburger A. (2005). Association of neurocognition, anxiety, positive and negative symptoms with coping preference in schizophrenia spectrum disorders. Schizophr. Res. 80, 163–171. 10.1016/j.schres.2005.07.005 16125370

[B48] Mäntylä T. Rönnlund M. Kliegel M. (2010). Components of executive functioning in metamemory. Appl. Neuropsychol. 17, 289–298. 10.1080/09084282.2010.525090 21154043

[B49] Maurer K. Zink M. Rausch F. Häfner H. (2016). The early recognition inventory ERIraos assesses the entire spectrum of symptoms through the course of an at-risk mental state. Early Interv. Psychiatry. [Epub ahead of print]. 10.1111/eip.12305 26801553

[B50] McGorry P. D. Nelson B. Amminger G. P. Bechdolf A. Francey S. M. Berger G.. (2009). Intervention in individuals at ultra-high risk for psychosis: a review and future directions. J. Clin. Psychiatry 70, 1206–1212. 10.4088/JCP.08r04472 19573499

[B51] Meisenzahl E. M. Schmitt G. J. Scheuerecker J. Möller H. J. (2007). The role of dopamine for the pathophysiology of schizophrenia. Int. Rev. Psychiatry 19, 337–345. 10.1080/09540260701502468 17671867

[B52] Moritz S. Woodward T. S. (2002). Memory confidence and false memories in schizophrenia. J. Nerv. Ment. Dis. 190, 641–643. 10.1097/00005053-200209000-00012 12357100

[B53] Moritz S. Woodward T. S. (2004). Plausibility judgement in schizophrenic patients: evidence for a liberal acceptance bias. Ger. J. Psychiatry 7, 66–74.

[B54] Moritz S. Woodward T. S. (2006a). Metacognitive control over false memories: a key determinant of delusional thinking. Curr. Psychiatr. Rep. 8, 184–190. 10.1007/s11920-006-0022-2 19817068

[B55] Moritz S. Woodward T. S. (2006b). The contribution of metamemory deficits to schizophrenia. J. Abnorm. Psychol. 115, 15–25. 10.1037/0021-843X.15.1.15 16492092

[B56] Moritz S. Gläscher J. Sommer T. Buechel C. Braus D. F. (2006b). Neural correlates of memory confidence. Neuroimage 33, 1188–1193. 10.1016/j.neuroimage.2006.08.003 17029986

[B57] Moritz S. Göritz A. S. Gallinat J. Schafschetzy M. Van Quaquebeke N. Peters M. J.. (2014b). Subjective competence breeds overconfidence in errors in psychosis. A hubris account of paranoia. J. Behav. Ther. Exp. Psychiatry. 48, 118–124. 10.1016/j.jbtep.2015.02.011 25817242

[B58] Moritz S. Goritz A. S. Van Q. N. Andreou C. Jungclaussen D. Peters M. J. (2014a). Knowledge corruption for visual perception in individuals high on paranoia. Psychiatry Res. 215, 700–705. 10.1016/j.psychres.2013.12.044 24461685

[B59] Moritz S. Veckenstedt R. Randjbar S. Vitzthum F. Woodward T. S. (2011). Antipsychotic treatment beyond antipsychotics: metacognitive intervention for schizophrenia patients improves delusional symptoms. Psychol. Med. 41, 1823–1832. 10.1017/S0033291710002618 21275083

[B60] Moritz S. Woodward T. S. Chen E. (2006a). Investigation of metamemory dysfunctions in first-episode schizophrenia. Schizophr. Res. 81, 247–252. 10.1016/j.schres.2005.09.004 16256310

[B61] Moritz S. Woodward T. S. Rodriguez-Raecke R. (2006c). Patients with schizophrenia do not produce more false memories than controls but are more confident in them. Psychol. Med. 36, 659–667. 10.1017/S0033291706007252 16512973

[B62] Moritz S. Woodward T. S. Ruff C. C. (2003). Source monitoring and memory confidence in schizophrenia. Psychol. Med. 33, 131–139. 10.1017/S0033291702006852 12537044

[B63] Moritz S. Woodward T. S. Cuttler C. Whitman J. C. Watson J. M. (2004). False memories in schizophrenia. Neuropsychology 18, 276–283. 10.1037/0894-4105.18.2.276 15099150

[B64] Moritz S. Woodward T. S. Jelinek L. Klinge R. (2008). Memory and metamemory in schizophrenia: a liberal acceptance account of psychosis. Psychol. Med. 38, 825–832. 10.1017/S0033291707002553 18205963

[B65] Moritz S. Woodward T. S. Whitman J. C. Cuttler C. (2005). Confidence in errors as a possible basis for delusions in schizophrenia. J. Nerv. Ment. Dis. 193, 9–16. 10.1097/01.nmd.0000149213.10692.00 15674129

[B66] Nelson T. O. Narens L. (1990). Metamemory: a theoretical framework and new findings, in The Psychology of Learning and Motivation, Vol. 26, ed Bower G. (San Diego, CA: Academic Press), 125–173.

[B67] Nuechterlein K. H. Green M. F. (2006). MCCB MATRICS Consensus Cognitive Battery. Los Angeles, CA: The Regents of the University of California.

[B68] Nuechterlein K. H. Barch D. M. Gold J. M. Goldberg T. E. Green M. F. Heaton R. K. (2004). Identification of separable cognitive factors in schizophrenia. Schizophr. Res. 72, 29–39. 10.1016/j.schres.2004.09.007 15531405

[B69] Ohtani T. Levitt J. J. Nestor P. G. Kawashima T. Asami T. Shenton M. E.. (2014). Prefrontal cortex volume deficit in schizophrenia: a new look using 3T MRI with manual parcellation. Schizophr. Res. 152, 184–190. 10.1016/j.schres.2013.10.026 24280350

[B70] Olsen R. K. Nichols E. A. Chen J. Hunt J. F. Glover G. H. Gabrieli J. D.. (2009). Performance-related sustained and anticipatory activity in human medial temporal lobe during delayed match-to-sample. J. Neurosci. 29, 11880–11890. 10.1523/JNEUROSCI.2245-09.2009 19776274 PMC2775810

[B71] Orenes I. Navarrete G. Beltrán D. Santamaría C. (2012). Schizotypal people stick longer to their first choices. Psychiatry Res. 200, 620–628. 10.1016/j.psychres.2012.03.030 22521898

[B72] Orfei M. D. Spoletini I. Banfi G. Caltagirone C. Spalletta G. (2010). Neuropsychological correlates of cognitive insight in schizophrenia. Psychiatry Res. 178, 51–56. 10.1016/j.psychres.2009.05.013 20452049

[B73] Perfect T. J. Schwartz B. L. (2002). Applied Metacognition. Cambridge, UK: Cambridge University Press.

[B74] Perlstein W. M. Carter C. S. Noll D. C. Cohen J. D. (2001). Relation of prefrontal cortex dysfunction to working memory and symptoms in schizophrenia. Am. J. Psychiatry 158, 1105–1113. 10.1176/appi.ajp.158.7.1105 11431233

[B75] Peters E. Joseph S. Day S. Garety P. (2004). Measuring delusional ideation: the 21-item Peters et al. delusions inventory (PDI). Schizophr. Bull. 30, 1005–1022. 10.1093/oxfordjournals.schbul.a007116 15954204

[B76] Peters M. Engel M. Hauschildt M. Moritz S. Jelinek L. Otgaar H. (2012). Investigating the corrective effect of forewarning on memory and meta-memory deficits in schizophrenia patients. J. Exp. Psychopathol. 3, 673–687. 10.5127/jep.022011

[B77] Peters M. Hauschildt M. Moritz S. Jelinek L. (2013). Impact of emotionality on memory and meta-memory in schizophrenia using video sequences. J. Behav. Ther. Exp. Psychiatry 44, 77–83. 10.1016/j.jbtep.2012.07.003 22925714

[B78] Rausch F. Eifler S. Esser A. Esslinger C. Schirmbeck F. Meyer-Lindenberg A.. (2013). The Early recognition inventory ERIraos detects at risk mental states of psychosis with high sensitivity. Compr. Psychiatry 54, 1068–1076. 10.1016/j.comppsych.2013.04.016 23759152

[B79] Rausch F. Eisenacher S. Elkin H. Englisch S. Kayser S. Striepens N.. (2016). Evaluation of the “Jumping to conclusion” bias in different subgroups of the at-risk mental state: from cognitive basic symptoms to UHR- criteria. Psychol. Med. 46, 2071–2081. 10.1017/S0033291716000465 27094404

[B80] Rausch F. Mier D. Eifler S. Fenske S. Schirmbeck F. Englisch S.. (2015). Reduced activation in the ventral striatum during probabilistic decision-making in patients in an at-risk mental state. J. Psychiatry Neurosci. 40, 163–173. 10.1503/jpn.140191 25622039 PMC4409434

[B81] Roediger H. L. III. McDermott K. B. (1995). Creating false memories: remembering words not presented in lists. J. Exp. Psychol. Learn. Mem. Cogn. 21, 803–814. 10.1037/0278-7393.21.4.803

[B82] Ruhrmann S. Schultze-Lutter F. Salokangas R. K. R. Heinimaa M. Linszen D. Dingemans P.. (2010). Prediction of psychosis in adolescents and young adults at high risk: results from the prospective european prediction of psychosis study. Arch. Gen. Psychiatry 67, 241–251. 10.1001/archgenpsychiatry.2009.206 20194824

[B83] Salavati B. Rajji T. K. Price R. Sun Y. Graff-Guerrero A. Daskalakis Z. J. (2015). Imaging-based neurochemistry in schizophrenia: a systematic review and implications for dysfunctional long-term potentiation. Schizophr. Bull. 41, 44–56. 10.1093/schbul/sbu132 25249654 PMC4266301

[B84] Scheyer R. Reznik N. Adres M. Apter A. Seidman L. J. Koren D. (2014). Metacognition in non-psychotic help-seeking adolescents: associations with prodromal symptoms, distress and psychosocial deterioration. Isr. J. Psychiatry Relat. Sci. 51, 34–43. 10.1016/S0920-9964(12)70471-1 24858633

[B85] Schneider B. C. Brune M. Bohn F. Veckenstedt R. Kolbeck K. Krieger E.. (2016). Investigating the efficacy of an individualized metacognitive therapy program (MCT+) for psychosis: study protocol of a multi-center randomized controlled trial. BMC Psychiatry 16:51. 10.1186/s12888-016-0756-2 26921116 PMC4769526

[B86] Sigurdsson T. Duvarci S. (2016). Hippocampal-prefrontal interactions in cognition, behavior and psychiatric disease. Front. Syst. Neurosci. 9:190. 10.3389/fnsys.2015.00190 26858612 PMC4727104

[B87] Slifstein M. van de Giessen E. Van Snellenberg J. Thompson J. L. Narendran R. Gil R.. (2015). Deficits in prefrontal cortical and extrastriatal dopamine release in schizophrenia: a positron emission tomographic functional magnetic resonance imaging study. JAMA Psychiatry 72, 316–324. 10.1001/jamapsychiatry.2014.2414 25651194 PMC4768742

[B88] Speechley W. J. Whitman J. C. Woodward T. S. (2010). The contribution of hypersalience to the ‘jumping to conclusions’ bias associated with delusions in schizophrenia. J. Psychiatry Neurosci. 35, 7–17. 10.1503/jpn.090025 20040242 PMC2799500

[B89] Veckenstedt R. Randjbar S. Vitzthum F. Hottenrott B. Woodward T. S. Moritz S. (2011). Incorrigibility, jumping to conclusions, and decision threshold in schizophrenia. Cogn. Neuropsychiatry 16, 174–192. 10.1080/13546805.2010.536084 21271413

[B90] Yung A. R. Yuen H. P. McGorry P. D. Phillips L. J. Kelly D. Dell'Olio M.. (2005). Mapping the onset of psychosis: the comprehensive assessment of at-risk mental states. Aust. N. Z. J. Psychiatry 39, 964–971. 10.1080/j.1440-1614.2005.01714.x 16343296

[B91] Zhou Y. Shu N. Liu Y. Song M. Hao Y. Liu H.. (2008). Altered resting-state functional connectivity and anatomical connectivity of hippocampus in schizophrenia. Schizophr. Res. 100, 120–132. 10.1016/j.schres.2007.11.039 18234476

